# Case Report: Dilated Cardiomyopathy in a Newborn, a Potential Association With SARS-COV-2

**DOI:** 10.3389/fped.2021.674300

**Published:** 2021-08-06

**Authors:** Estela Azeka, Adam Arshad, Cristiane Martins, Anna Claudia Dominguez, Adailson Siqueira, Andre Silveira Loss, Marcelo Jatene, Nana Miura

**Affiliations:** ^1^Heart Institute (InCor), University of São Paulo Medical School, São Paulo, Brazil; ^2^BioCor Instituto, Belo Horizonte, Brazil

**Keywords:** COVID-19, newborn, heart failure, heart transplantation, dilated cardiomyopathy

## Abstract

**Objective:** The objective of this study was to describe the clinical course of a newborn who developed dilated cardiomyopathy (DCM) after COVID-19 infection.

**Methods:** We retrospectively assessed the clinical notes of a pediatric patient with decompensated heart failure and who was previously positive for severe acute respiratory syndrome coronavirus 2 (SARS-CoV-2).

**Results:** A 23-day-old newborn presented with diarrhea, hypoactivity, tachypnea, and lethargy. The infant progressed to develop respiratory failure and required orotracheal intubation due to apnea. A nasopharyngeal swab tested positive for SARS-COV-2. An echocardiogram (ECHO) demonstrated severe left ventricular dysfunction. The patient was discharged after 18 days with furosemide and angiotensin-converting enzyme inhibitors. During the follow-up period, the infant had two episodes of decompensated heart failure, with evidence of DCM. Investigations for known causes of secondary DCM were negative. The infant was promptly referred for heart transplantation.

**Conclusion:** Although rare, we have observed a case of DCM in a newborn following COVID-19 disease. DCM may be a complication following COVID-19 disease in newborns.

## Introduction

Coronavirus disease 2019 (COVID-19) is a global pandemic affecting over 151 million people worldwide and carrying an ~1.7% mortality rate. São Paulo is considered a hotspot for the disease, with 2.89 million confirmed cases and over 95,000 deaths within the state to date (1, 2).

A number of small reports indicate that children are just as likely as adults to be infected by the virus. Although it is considered that children have less severe clinical symptoms, the potential harm of this novel disease remains largely unknown in neonates (3, 4). Given the deleterious effects of several respiratory viruses on newborns with developing immune systems, delineating potential consequences of this viral infections of importance.

This report provides our center's experience, as a tertiary pediatric cardiac center in a developing nation, in managing an infant who developed dilated cardiomyopathy (DCM) after COVID-19 disease. This will help add to the evidence base on the potential implications of COVID-19 on the newborn population, as well as appropriate management strategies.

## Case

A 23-day-old, female, term newborn (birth date: September 03, 2020) was admitted to a district general hospital in Belo Horizonte, Brazil. The infant was hypothermic, dehydrated, and hypoactive, with diarrhea, vomiting, and poor peripheral perfusion. There was no relevant obstetric or developmental history. The infant's birth weight was 3.250 g, and their height was 48 cm. The infant was immediately referred to the intensive care facility for appropriate monitoring and investigations, including X-rays, blood tests, and a lumbar puncture (LP). These investigations demonstrated no significant abnormalities (Table 1).

**Table 1:** Laboratorial results during the patient's admission.

**Test**	**Results**
Blood cultures	Negative
Cerebral spinal fluid	Negative
Viral panel	Negative
**RT-PCR COVID 19**	Positive
Blood cultures	No growth
**Screening for:**	
Phenylketonuria	Negative
Congenital hypothyroidism	Negative
Sickle cell anemia	Negative
Cystic fibrosis	Negative
Biotinidase deficiency	Negative
CAH	Negative
G6PD	Negative
AMD	Negative
**Laboratory results**	
D-dimer	5,437
Troponin	0.55
CKMB	185
PCR	32 (<5 mg/l)
proBNP	>30.000
Leukocytes	17.300
Chest CT scan	Bilateral pneumothorax, without pneumonia
Ammonia	72.7 μl/l (18–72 μl/l)

*Positive values are highlighted in bold. CAH, congenital adrenal hyperplasia; PCR, polymerase chain reaction; AMD, amino acid metabolism disorders*.

The patient developed significant apnea episodes and requiringoral intubation with mechanical ventilation. A chest CT scan was conducted, which demonstrated bilateral pneumothoraxes. A drain was inserted to treat the pneumothoraxes, resolving the infant's respiratory distress. The initial chest X-ray had a normal cardiac size, and the CT scan showed no signs of frosted glass. A COVID-19 PCR test was conducted and was positive. The parents were not positive for COVID-19, albeit one of the caregivers (the father) worked as a physician and had contact with COVID-19 patients. An echocardiogram (ECHO) showed an ejection fraction (EF) of 49% (moderately reduced) with no other significant abnormalities (Supplementary Table 2).

It was considered that the patient had viral myocarditis and was treated with a fluid infusion, dobutamine, epinephrine, and antibiotics for 10 days (cephalosporins, beta-lactam antibiotics, and aminoglycosides). The infant was weaned from mechanical ventilation and discharged from the hospital for outpatient follow-up at 18 days with furosemide and an angiotensin enzyme inhibitor (ACEi).

At 55 days of life (on October 27, 2020), the infant returned to the hospital, presenting with tachypnea, tachycardia, lethargy, diarrhea, vomiting, and difficulty in breast feeding (Supplementary Table 1). Their symptoms were similar to their last admission to the hospital. The infant required intubation with mechanical ventilation and developed clinical signs of decompensated heart failure with worsening left ventricle function, by ECHO. The patient received dobutamine, epinephrine, and levosimendan. Given that the provisional diagnosis was a post-viral cardiomyopathy, immunoglobulin was administered. In total, the child was hospitalized for 55 days, being discharged at 3 months of age with spironolactone, ACEi, carvedilol, furosemide, and hydralazine.

On December 30, 2020 (age: 3 months), the infant presented with signs of low output (which included emesis, cold and sticky skin, drowsiness, tachypnea, and tachycardia). During an attempt to pass a PICC line, the child became increasingly agitated with apneic episodes. A COVID PCR was negative for COVID-19. The infant was intubated with mechanical ventilation for 2 days, and CPAP for a further day. During this period, the infant received vasoactive drugs (milrinone, dobutamine, and epinephrine). An ECHO showed severe DCM with mitral valve insufficiency, pulmonary hypertension, and an EF of 35%.

Given the concerning emerging features, the infant was transferred to a tertiary cardiac center in São Paulo on February 12, 2021. On transfer, the patient was receiving mechanical ventilation, with epinephrine 0.06 mcg/kg/min and milrinone 1 mcg/kg/min. A prompt cardiac work-up was conducted, including two ECHO scans:

1. The first was conducted on February 12, 2021, on admission. This demonstrated DCM with pulmonary hypertension and left ventricular systolic dysfunction (LVEF) of 28%.
2. The second ECHO was conducted on April 29, 2021. This demonstrated worsening systolic function, LVEF 17%.

**Figure 1:**
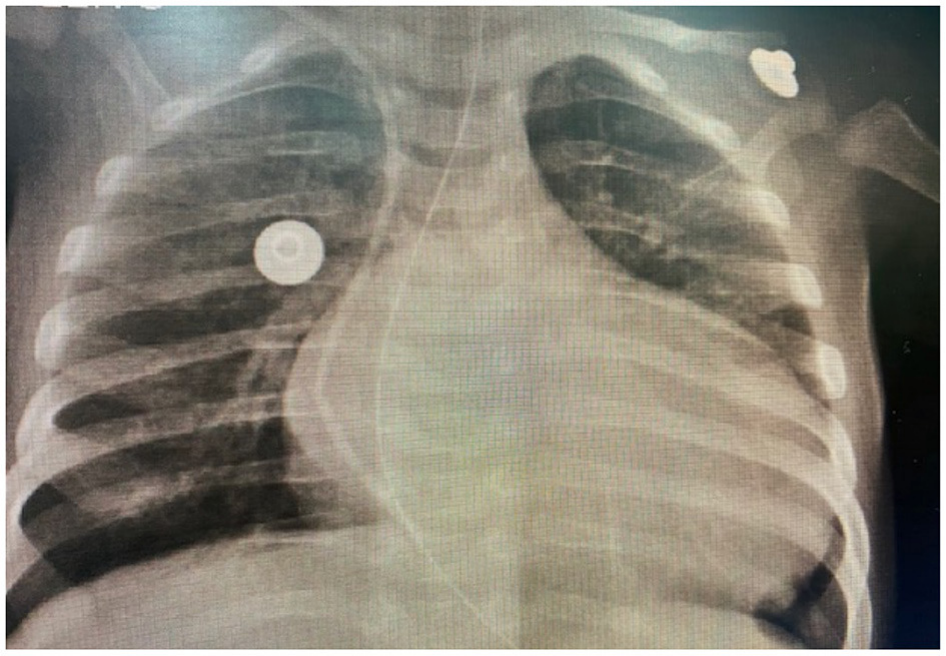
Chest X-ray of the patient, taken on the February 12, 2021.

A CXR demonstrated significant cardiomegaly (Figure 1). The patient was negative for Coxsackie virus and Parvovirus. A gallium-67 scintigraphy was negative for an active cardiac inflammatory process. An abdominal ultrasound was unremarkable. A Holter demonstrated normal sinus rhythm (max: 195, min: 86, average: 129, EEVV: 27 EEAA: 21). A thorough evaluation excluded innate errors of metabolism as well as major genetic causes, with whole-genome sequencing demonstrating a variant of uncertain significance which was not known to cause ventricular dysfunction. There was no family history of cardiomyopathy, arrhythmia, or sudden cardiac death. Finally, a cardiac MRI demonstrated left ventricle DCM without late enhancement or edema and a discrete pericardial effusion (EF 21%, left ventricle end diastolic diameter: 40 mm, left ventricle end systolic diameter: 36 mm). Serologies for myocarditis were negative. With difficulties in weaning the infant from inotropic drug therapy, the patient was promptly referred for pediatric cardiac transplantation.

## Discussion

The COVID-19 outbreak has led to unique challenges in appropriate patient management. The disease, while primarily affecting the lungs, has a spectrum of presentations ranging from asymptomatic to viral pneumonia and respiratory failure, and in children, multisystem inflammatory syndrome in children (MIS-C) (5). As our understanding of this condition continues to improve, its complex multisystem effects are being revealed.

Our case demonstrates a patient with DCM in the setting of a positive COVID swab. Appropriate diagnostic screening failed to identify an underlying, known precipitant for this presentation, including bacterial, viral, parasitic, endocrine, and autoimmune, which have been detailed within the literature. It is with this reason that we present our above case, to add to the literature of a potential association between COVID-19 and DCM.

Dilated cardiomyopathy is a condition characterized by the enlargement and dilatation of one or both ventricles, alongside impaired contractility (with a LVEF < 40%). We classify its disease process as primary or secondary; primary DCM is considered idiopathic, and the diagnosis can only be made after excluding secondary causes. Most significant is the genetic associations with DCM, with one study demonstrating gene mutations in the TTN gene being responsible for 25% of familial cases of idiopathic DCM and 18% of sporadic cases (6). The average age of presentation is 14 months among children. In most cases DCM is progressive, leading to heart failure and death. Without a transplant, the survival rates are poor (7, 8).

We appreciate that our finding may be entirely incidental—a positive COVID result in a patient independently at risk of developing DCM from alternative pathophysiological mechanisms. The sensitivity of our PCR methods in COVID-19 detection are 70 and 95%, respectively (9). However, the persisting clinical signs of cardiogenic shock and worsening ventricular function, in exclusion of secondary causes and a negative family history, arises our suspicions of a potential COVID-related pathogenesis. With our rapidly evolving understanding of this viral infection, we believe our case provides valuable information as to a potential association between COVID-19 and DCM.

The association between COVID-19 and cardiovascular pathologies is detailed within the adult literature, including acute myocardial infarction, myocarditis, cardiomyopathy, arrhythmias, and venous thromboembolism. Specific to cardiomyopathy, rare cases of COVID-19-associated Takotsubo cardiomyopathy have been described in the adult literature, often presenting with ECG changes including ST elevation or marked T-wave inversion, concerning for ACS (10).

Several pathophysiological mechanisms may underpin how severe COVID-19 disease may contribute to the development of DCM. The COVID-19 virus binds to the ACE2 receptor, which is expressed on cardiac myocytes and vascular endothelium. Furthermore, severe COVID disease is known to trigger a cytokine storm, with increased levels of IL1B, IFNγ, GCSF, MIP1A, and TNFα (11). Finally, a stress response in relation to respiratory failure, hypoxia, and shock will trigger an increase in cortisol and catecholamines. The combination of all the above factors may precipitate the development of DCM.

Our case further provides a verbal description of the challenges in managing pediatric heart disease in the context of the COVID-19 pandemic, and when healthcare services are significantly strained in Brazil. Our center is the largest cardiac transplantation unit in the nation; however, dealing with an increasingly unwell population and with a reduced bed capacity has led to an increased shift to Telehealth (and reduced physical interactions with patients). This creates significant challenges for patient care.

Overall, our case demonstrates a child with persistent signs of decompensated heart failure after COVID-19 viremia, with the development of DCM. Secondary causes were promptly excluded, with negative genome sequencing. This may demonstrate an association between COVID-19 disease and DCM.

## Conclusion

COVID-19 infection in newborn may evolve with severe cardiogenic shock and DCM. Heart transplantation might be a therapeutic option.

## Data Availability Statement

The raw data supporting the conclusions of this article will be made available by the authors, without undue reservation.

## Ethics Statement

The study was approved by the Ethics Committee at Heart Institute (InCor), University of São Paulo Medical School. The legal guardian provided written informed consent to participate in this study.

## Author Contributions

EA, AA, CM, AD, AS, AL, MJ, and NM: designed study and data extraction. EA and AA: data analysis, data interpretation, and wrote original draft. All the authors reviewed manuscript, contributed to the article, and approved the submission version.

## Conflict of Interest

The authors declare that the research was conducted in the absence of any commercial or financial relationships that could be construed as a potential conflict of interest.

## Publisher's Note

All claims expressed in this article are solely those of the authors and do not necessarily represent those of their affiliated organizations, or those of the publisher, the editors and the reviewers. Any product that may be evaluated in this article, or claim that may be made by its manufacturer, is not guaranteed or endorsed by the publisher.

## Abbreviations

COVID 2019: coronavirus disease 2019
CRP: C-reactive protein
ECHO: echocardiogram
EF: ejection fraction
SARS-CoV-2: severe acute respiratory syndrome coronavirus 2
RT-PCR: reverse-transcriptase polymerase chain reaction.
