# Supplementary material for: Case Report: Dilated Cardiomyopathy in a Newborn, a Potential Association With SARS-COV-2
Source: Front Pediatr. 2021 Aug 6;9:674300. doi: 10.3389/fped.2021.674300 (PMC8377194; doi:10.3389/fped.2021.674300)
Supplement: Supplementary file 1 [file Table_1.DOCX]

**Supplementary Appendix**

**TABLE 1. TABLE DEMONSTRATING THE DATES FOR THE 1^ST^ AND 2^ND^ ADMISSIONS**

|  |  | First Admission |  | Second admission |  |  |  |
| --- | --- | --- | --- | --- | --- | --- | --- |
|  |  | Dates |  | Dates |  |  |  |
|  |  | 09/25/2020 to10/13/2020 |  | 10/27/2020 to12/23/2020 |  |  |  |
| Mechanical ventilation |  | 09/25/2020 to10/7/2020 |  | 10/27/2020- to11/04/2020 |  |  |  |
| Vasoactive drugs |  | 09/25/2020 to10/07/2020 |  | 10/27/2020 to11/19/2020 |  |  |  |

**TABLE 2. ECHOCARDIOGRAM RESULTS FOR THE PATIENT**

| Date | Ejection Fraction (EF) % |
| --- | --- |
| 09/25/2020  10/01/2020 | 49 with dobutamine  42 |
| 10/05/2020 | 55 |
| 10/09/2020 | 59 with discrete left ventricle dilation, mitral regurgitation, receiving angiotensin-converting enzyme inhibitor and furosemide. |
| 10/27/2020 | 38 |
| 10/30/2020 | 39 with moderate to important mitral insufficiency |
| 11/3/2020 | 46 with important left ventricle dilation |
| 11/10/2020 | 38 severe left ventricle dilation |
| 11/20/2020 | 50 (after levosimendan) with left ventricle dilation |
| 11/24/2020 | 42 with moderate to severe left ventricle dilation |
| 11/27/2020 | 46 with moderate to severe left ventricle dilation |
| 12/4/2020 | 56 with moderate to severe left ventricle dilation |
| 12/10/2020 | 46 with moderate to severe left ventricle dilation |
| 12/14/2020 | 49 (moderate to important dilation of left ventricle: DDVE 27 mm) |
| 12/18/2020 | 50 (important left ventricle dilation DDVE: 30mm) |
| 12/23/2020 | 46 with moderate to severe left ventricle dilation |
